# Hospital and Institutional News

**Published:** 1918-01-05

**Authors:** 


### HOSPITAL AND INSTITUTIONAL NEWS.

### HOSPITAL DEVELOPMENT IN THE U.S.A.

The administration of American hospitals has developed greatly since 1905 especially. Up to that date hospital administration of the highest type was mostly in its infancy, with the exception of a relatively few great hospitals in the Eastern States, which deservedly had a world-wide reputation. Since then the people throughout the United States, with few exceptions, have become alive to the importance of hospitals of maximum efficiency under the direction of a superintendent, usually a member of the medical profession, in a few cases a layman, and here and there a woman, who constitute brilliant exceptions. The net result has been remarkable extensions and improvements in hygienic conditions, administrative efficiency, the development of special departments, and advances in treatment, in the commissariat, economy, equipment, and discipline. The training of nurses has been developed too, and the nurse-training schools have attracted an increasing number of educated gentlewomen, many of whom have taken degrees at a recognised university before entering upon their training.

### A MEDICAL SUPERINTENDENT AND A SECRETARY?

Elsewhere in the present issue we call attention to the outlook in regard to hospital administration in the United Kingdom at the present time, and the changes which are bound to come after the war. In this connection it is worthy of note that the regulations governing the duties and emoluments of the new secretary to King's College Hospital, to be appointed to fill the vacancy caused by the lamented death of Captain Tunnard, charge the secretary with "the immediate superintendence . . . of all Resident Officers whilst within the hospital," the obtaining and collecting of funds, including "the provision of money from every available source for the needs of the hospital and convalescent home" which "must occupy his constant attention," the issue of special appeals, and the enforcement of the observance throughout the establishment of the bye-laws and regulations and orders of the committee, in addition to a multiplicity of routine duties which alone cover ground sufficiently extensive to occupy fully the whole time of a business secretary. These include the economical management of the hospital in every department—a matter for his unceasing care—and the duty of superintendent almoner, with serious responsibilities attaching to the admission and discharge of the patients. He is to keep on friendly terms with the resident medical officers, and to preside at their table. Candidates up to the age of fifty on the day of their application are eligible, but no retiring allowance attaches to the office. A perusal of the conditions of the office and its duties compels the experienced administrator to conclude that the emoluments, even to approach an adequate sum, could not be less than £1,000 per annum. In fact the salary offered is to commence at £400, rising in annual increments of £25 to a maximum of £550, with board and a house, or its equivalent in money.

### WHAT IS THE EXPLANATION?

Contrast this with the American system, where the secretary is usually responsible for the finances and business side of the administration, with a medical superintendent in responsible control of the *personnel* and general administration of the hospital proper in every department, with a salary of £1,500 to £2,000 a year. Is the explanation to be found in an intention of the chairman or some other governor himself to undertake in an honorary capacity the actual direction of affairs, including the raising of the necessary funds? How otherwise can King's College Hospital Committee expect to find a fully trained administrator, of wide experience, resting upon past services for a great hospital, which have won for him reputation and authority in the hospital world? No other man or woman could be adequately qualified, even providing the successful candidate be a colossal worker of robust health, willing to undertake cheerfully the enormous mass of detail and the widespread responsibilities set forth in the list of duties, which few, if any, men not already occupied and better paid, are competent to fulfil.

### THE NEW YEAR HONOURS

The New Year Honours contain names well known in the professions of medicine and surgery, in the field of charity and in that of philanthropy. Of the former appear in the main list Dr. Barclay Baron, M.B., C.M., consulting physician to the throat and nose department of the Bristol General Hospital, an ex-president of the British Medical Association, and the Lord Mayor of Bristol, who has been made a knight. The same honour has been conferred on Mr. T. J. Horder, M.D., F.R.C.P., assistant physician to St. Bartholomew's Hospital and physician to the Cancer Hospital; Mr. John Phillips, M.D., F.R.C.P., consulting obstetric physician to King's College Hospital and the British Lying-in Hospital, and Emeritus Professor of Obstetric Medicine at King's College; and on Mr. Harold J. Stiles, M.B., C.M., F.R.C.S., surgeon to the Royal Edinburgh Hospital for Sick Children and the Chalmers Hospital, Edinburgh. Mr. Almeric H. Paget, whose name is well known in the world of charity in connection with the Military Massage Corps which he and the late Mrs. Paget founded at the outbreak of war, which has been carried on with increasing success ever since, has been made a baron; Major Sir Henry Norman, Bt., M.P., who with Lady Norman established the British Hospital at Wimereux, has been made a Privy Councillor; Mr. John Leigh, who has given munificently to provide institutional treatment for disabled soldiers and for the treatment of soldiers suffering from shell-shock, has been made a baronet. Mr. Leigh has also given a memorial hospital at Altrincham to the British Red Cross Society for the use of 100 wounded soldiers, which after the war he intends to carry on as the Cheshire County Home for sailors and soldiers totally disabled during the war. Mr. David S. Davies, treasurer of the King Edward VII. Welsh National Memorial Association, has been made a knight, and the same honour has been conferred on Mr. W. H. Seager, of Cardiff, who has contributed largely to King Edward VII.'s Hospital, Cardiff, and other institutions; Mr. Howard Kingsley Wood, deputy chairman of the London Pension Authority and chairman of the London Insurance Committee; and Mr. James Bird, clerk to the London County Council since 1913.

### NAVAL AND MILITARY HONOURS.

Surgeon-Generals W. H. Norman, C.B., R.N., and George L. Cheatle, C.B., C.V.O., F.R.C.S., R.N., have been appointed Knight Commanders of the Most Honourable Order of the Bath (Civil Division) in recognition of services during the war, whilst Surgeon-Generals Arthur Edmunds, F.R.C.S., R.N., William W. Pryn, R.N., James Lawrence Smith, M.V.O., R.N., and Deputy Surgeon-General D. J. P. McNabb, R.N., have been appointed Companions of the same Order. Staff Surgeon Henry Cooper, B.A., R.N., has been awarded the Distinguished Service Order. In the Military Division of the Order of the Bath the following have been appointed Companions for valuable services rendered in connection with military operations in the field: Surgeon-General R. H. S. Sawyer, C.M.G., M.B., Col. R. H. Firth, F.R.C.S., Col. and Hon. Surg.-Gen. B. M. Skinner, C.M.G., M.V.O., Lt.-Col. and Bt. Col. (T. Col.) Frederick Smith, C.M.G., D.S.O., Surg.-Gen. H. N. Thompson, C.M.G., D.S.O., M.B., Cols. A. J. Luther, J. M. Forrest Shine, J. B. Wilson, C.M.G., M.D., F. R. Newland, C.M.G., M.B., and H. C. Thurston, C.M.G., T. Cols. A. H. Tubby, C.M.G., M.B., F.R.C.S., and S. Maynard Smith, M.B., F.R.C.S., Lt.-Cols. C. J. Trimble, C.M.G., A. R. Aldridge, C.S.I., C.M.G., M.B., and T. F. Dewar, Col. A. D. Sharp, C.M.G., F.R.C.S., Col. Alfred Sutton, C.M.G., Australian Army Medical Corps, and Col Chas. Mackie Begg, C.M.G., M.D., F.R.C.S., of the New Zealand Medical Corps. In the Order of St. Michael and St. George Surgeon-General Sir G. H. Makins, K.C.M.G., C.B., F.R.C.S., has been promoted to G.C.M.G., and the following other promotions or appointments have been made for services rendered in connection with military operations in the Field:—*K.C.M.G.*—Surgeon-General W. G. Macpherson, C.B., C.M.G., M.B., Col. and Hon. Surg.-Gen. J. Murray Irwin, C.B., M.B., Col. and Hon. Surg.-Gen. James Maher, C.B., T. Col. J. Purves Stewart, C.B., M.D., F.R.C.P., T. Col. Thos. Crisp English, C.M.G., M.B., F.R.C.S. *C.M.G.*—Cols. P. C. H. Gordon, C. J. MacDonald, M.D., E. G. Browne, C.B., and S. G. Moores, C.B., Lt.-Col. and Bt. Col. (T. Col.) Howard Ensor, D.S.O., M.B., T. Col. Wm. Pasteur, M.D., Lt.-Col. Chas. T. Hudson, I.M.S., Lt.-Col. (T. Col.) D. D. Shanahan, Cols. E. W. Bliss, D.S.O., and A. E. C. Keble, D.S.O., T. Hon. Lt.-Col. Nathan Raw, M.D., Col. C. W. Profeit, D.S.O., M.B., Col. R. J. Blackham, C.I.E., D.S.O., T. Lt.-Cols. E. H. Starling, M.D., F.R.C.P., and L. S. Dudgeon, F.R.C.P., Lt.-Col. (T. Col.) A. T. White, Lt.-Col. C. I. Ellis. For services rendered in connection with the war the following promotions in or appointments to the Most Honourable Order of the Bath have been made:—*K.C.B.* (Military Division).—Surg.-Gen. Sir David Bruce, C.B., F.R.S., F.R.C.P. *C.B.* (Military Division).—Surg. Gen. F. J. Jencken, M.B., T. Col. H. H. Tooth, C.M.G., T. Hon. Lt.-Col. G. S. Buchanan, M.D., T. Col. Andrew Balfour, C.M.G., M.D., F.R.C.P. In recognition of valuable services in connection with the war the following promotions to or appointments in the Most Distinguished Order of Saint Michael and Saint George have been made:—*K.C.M.G.*—T. Col. A. E. Garrod, C.M.G. M.D., F.R.S., T. Col. C. A. Ballance, C.B., M.V.O., M.B., F.R.C.S. *C.M.G.*—Cols. Jos. Griffiths, M.D., F.R.C.S., C. P. Oliver, M.D., and C. H. Melville, M.B., T. Hon. Col. Sir John Collie, M.D., T. Hon. Lt.-Cols. H. R. Kenwood, M.B., J. Robertson, M.D., T. Lt.-Cols. J. C. G. Ledingham, and C. C. M. Wenyon, M.B., T. Lt.-Col. G. B. Price, M.D., Lt.-Col. H. S. Anderson, Maj. T. W. Griffith, M.D., Lt.-Col. W. M. Roocroft; and of the Australian Medical Corps, Lt.-Col. John Gordon, Lt.-Col. (T. Col.) D. M. McWhae, Hon. Lt.-Col. J. A. Murdoch, and Lt.-Col. (T. Col.) Kenneth Smith.

### GUARDIANS AND THE CLAIMS OF THE HOSPITALS.

Southwark Board of Guardians have decided to adjourn for six months the question as to whether they shall subscribe to the funds of various hospitals. The matter arose out of an appeal from Lord Sandhurst, treasurer of St. Bartholomew's Hospital, who pointed out that not only were they treating a large number of patients from Southwark at the hospital, but that the governors, irrespective of leasehold property in the borough, contributed yearly towards the rates £1,025 in respect of weekly properties. It was at once pointed out that if St. Bartholomew's Hospital had a claim upon Southwark, Guy's Hospital had even a greater claim, inasmuch as whilst a hundred Southwark patients might be treated at Bart.'s, thousands were treated at Guy's. Then naturally came the claims of King's College Hospital, where scores of patients from the northern end of the borough go. In quick succession the Evelina Hospital for sick children, the Belgrave Hospital for Children, the General Lying-in Hospital, York Road, and the various hospitals dealing with special diseases were put forward as worthy of consideration. It was in the multitude of claims that the Guardians came to an *impasse*, and they felt they could not deal with the matter at the present time. There is little doubt, however, that when the board take this question in hand they will deal fairly and generously with the claims of all the hospitals in their area.

### THE REGISTRATION OF ALIENS.

A point of some importance to hospitals has been brought to the notice of the secretary of a special hospital in London by a visit from a police officer, who called "to inspect the Register of Aliens." It appears that hospitals which admit paying patients must comply with the Home Office regulations regarding aliens in the same way as do hotels and boarding-houses. It is obvious that aliens might easily be lost sight of by the police on their admission to hospital, but the present application of the regulation appears to be ludicrously insufficient. The police are acting in accordance with the strict letter of the law, which requires registration only in the case of persons received "for fee or reward." Yet, obviously, the number of aliens received by hospitals as paying patients is insignificant compared with the number received as free cases. Moreover, the use of an ambiguous expression like "paying patient" is fraught with difficulties. As generally understood, a paying patient is one who reimburses to the hospital the whole, or a substantial part, of the cost of his maintenance. Yet in the particular instance under notice, the patients pay (if anything) only 5s. a week. They are thus "paying" in a literal sense, and the police apparently interpret the regulations in that precise manner. For the benefit of secretaries, it may be explained that the "Register of Aliens" need not be specially printed or prepared, but that each alien inmate should fill up on admission an appropriate form giving full particulars of himself. These forms may be obtained at the Post Office. It is to be hoped, however, that if the authorities insist upon a close adherence by hospitals to the regulations, the regulations themselves may be amended so that they shall no longer be stultified by the very insufficiency of their application.

### A BOOK OF APPEALS.

It would strike terror into the heart of many secretaries if it were proposed that every appeal issued on behalf of each hospital during a year should be bound up in book form at the end of December, and sent out in January *as likely to influence the general public.* Too many of these appeals are so perfunctory and so appallingly dull that it would not be a bad thing if their authors were compelled to read them out loud to a mixed audience in a picture hall which had expected to witness an exciting film upon the "secrets of the operating theatre." The reading would empty the hall as quickly as a fire. Yet there is nothing but intellectual laziness at the bottom of this rubbish. We have Mr. Rea writing letters in the Cardiff papers ranging from a column in length to two words, and we hear that some have been popular—think of it: an appeal popular! Mr. Rea is like an editor with his mind always intent on finding illustrations for his thesis; and he cannot walk down a side street with his little child, or see a queue waiting for the second house without finding suggestions for future articles. In fact he has become one of the most varied writers on the Cardiff press, and we hope next to see him engaged on a weekly "hospital causerie." The reprinting of his articles would form a standard for this class of hospital appeals.

### WAR SAVINGS IN SCOTLAND.

When a carefully prepared campaign of thrift, such as that used by the Scottish War Savings Committee, was launched in a thrifty country such as Scotland, a certain element of success could be foreseen in the result. The committee must have reason to be pleased with the success of the scheme, if they are not surprised at the widespread popularity of this method of saving small sums and at the same time helping to finance the war. The figures published in the first annual report of the committee are substantial and gratifying; during the first six months of the current year (the work did not get properly under way until December 1916) the cash value of certificates sold was £4,123,000. It is pleasing to read that the movement in munition and controlled establishments, where 319 associations have been formed, has attracted the workers, and that at least part of the good money they are now earning will be available when the inevitable rainy day arrives.

### ART AND SPORT AIDING CHARITY.

One of the brightest and best developments of art and sport in modern times is the close association of those services with the welfare and support of charitable institutions. Music and the drama throughout the ages have invariably been bound up with charity and philanthropy, and the exponents of these ancient arts have proved pillars of support in all great movements for the welfare of the community. Modern sport has, to the great credit of its participants and supporters, also linked itself closely with charitable effort. Two instances of this happy association are forthcoming in the endowment of two cots in the Leicester Children's Hospital. The Music and Dramatic Society of the town have in past years, by accumulative donations, permanently endowed one cot at a cost of £500, and they have recently paid to the institution a further sum of £140 towards a second cot, bringing their total contributions to £880. They hope to complete the second endowment in the New Year. The ancient game of bowls claims many supporters in the Midlands, and the bowling clubs of Leicester and the county have recently put into operation a movement for the endowment of a children's cot. Their initial effort has been successful, £25 having been sent for the annual endowment for one year, and they hope not only to maintain this for the future, but gradually to provide for the permanent endowment. The Hospital has always urged the great field which is open to those institutions which are alert enough to seek out the opportunities which lie open to their needs.

### THE LATEST GAZETTE.

It was surely time that Queen Alexandra's Hospital for Officers should produce its own gazette, and we welcome the first number, which was, indeed, the Christmas one. Among the contributors is a "regular" writer to *Punch*, and this sort of talent still lurks so often in hospitals which run no gazette that it is worth while to mention the discovery at this Highgate institution. The "old house" accommodates the nurses; the officers live in the new temporary wooden wards which were designed by Mr. Claude Ferrier, F.R.I.B.A. There are thirty-three beds at Highgate, and others at the annexe in Portland Place and elsewhere. The magazine consists largely of verse, in which one contributor, "A. P. H.," shows a genuine talent. His poem, "The Atrocity," has that peculiar tang about it which makes truth recognisable whenever it is written. On the refrain "The A.D.C. has got the Cross" he writes verses which deserve a place in any anthology of the war. They are so unmistakably sincere, which is more than can be said of most of the war poems which have been published. We hope that he will write again.

### A BETRAYAL OF TRUST.

Advertisements and articles face each other on alternate pages with such regularity in the latest number of the gazette of the 4th Southern General Hospital, Plymouth, that it is rather a puzzle to find the articles. There are a few illustrations and drawings; moreover, the advertisements are on the right-hand page, in consequence of which in despair at escaping from them there is a great temptation to throw the whole paper into the waste-paper basket. It really is absurd to overdo advertisements in this way. One neither reads them nor the articles which peep out between. To make a magazine unreadable is not the way to edit it. If advertisers wish to publish a gazette let them do so, but to do so by using the name of a great hospital is almost a betrayal of trust on the part of those who have permitted it.

### HUN PROFESSORS IN AN AIR-RAID

The German mind has always been interested in questions of morbid psychology, and a professor in the University of Freiburg has recently published an article in the *Medizinische Klinik* of Berlin (quoted in the current number of the *British Medical Journal*), giving an account of the effects of our bombs upon the local population. These effects were apparently directly proportional to the loudness of the explosions; thus the earlier raids, when bombs of small calibre were dropped, made but little impression, but in the later attacks the terrific noise of the explosions so frightened, to take a single example, an artillery officer that he stated he was far more alarmed by them than by the bombardment on the Somme. The sound made by the falling bombs preceded the explosions by several seconds, and this period of suspense had a truly terrifying effect, "the most common exhibitions of fear being chattering of the teeth, pallor, more or less mechanical praying, hysterical laughter, diarrhœa, rapid excretion of urine, and great thirst." The raids, however, did not provide the asylums with a single new case, and the inmates of the Freiburg Asylum were little disturbed, and even regarded the bombardment as "an agreeable entertainment." This observation bears out in an interesting way the experiences at a London institution which narrowly escaped in a recent raid. We trust that our airmen will continue their vigorous and impressive visitations.

### THE V.A.D. IN DEVONSHIRE.

Devon's great share in the national work of the V.A.D. is emphasised in a report issued recently by Mr. J. C. Davis and Mr. A. W. Trethewey, the county director and county secretary respectively of the V.A.D. in Devonshire. They point out with pardonable pride that of the 60,000 beds which have been provided in Red Cross and St. John hospitals in England and Wales up to the end of May last one-twentieth have been found by their county, although the population of Devon is only one-fiftieth of the larger area, and that their cost of maintenance is less than 4s. per head. The report, which runs to sixty interesting pages, pays a high tribute to the devoted work of the women in the hospitals, which, it says, is beyond all praise.

### THIS WEEK'S DRUG MARKET.

As the process of stocktaking is hardly completed as yet, only such buying as is necessary to cover immediate requirements is taking place. But we may expect to see business fully resumed before the month is half over, for in these times the market is never inactive for many days together. It is not at all improbable that quite early in the year we shall find the Government control of drugs becoming gradually tighter, for as supplies become scarcer it will be necessary to ensure a reasonably just distribution of them and an entire absence of hoarding and speculation. There is no reason whatever to believe that the general upward movement which has prevailed since the beginning of the war is going to stop before the war is over. Several of the synthetic drugs may be exceptions to this rule, since the output of British factories is increasing in salicylic acid, sodium salicylate, phenacetin, and aspirin. Phenacetin has started the year with a distinctly lower price, and as regards the salicylates, in spite of the falling off in supplies from the United States, there is no scarcity. Balsam Tolu is dearer. Potassium bromide crystals are by no means in good supply, and the price may advance. As was not unexpected, the prices of rectified spirit, mineralised methylated spirit, and industrial spirit have been advanced. This will entail a rise in the prices of spirituous preparations, and of those preparations in the manufacture of which spirit is used. Quotations for chloroform and ethers have been raised. Manufacturers of lithia carbonate and lithia citrate have advanced their quotations. Almond oil is a trifle dearer.

